# Benzovindiflupyr Is Associated with Metabolic Homeostasis Disturbance and Gut–Liver Axis Alterations in Zebrafish: Insights from a Multi-Omics Approach

**DOI:** 10.3390/ijms27125455

**Published:** 2026-06-17

**Authors:** Jiyan Miao, Shihang Han, Xinrui Dang, Qi Chen, Jinling Diao, Wentao Zhu

**Affiliations:** Innovation Center of Pesticide Research, Department of Applied Chemistry, College of Science, China Agricultural University, Beijing 100193, China; miaojiyan@cau.edu.cn (J.M.); zlwdmiao@126.com (S.H.); 15600912258@sohu.com (X.D.); zlwdmayo@gmail.com (Q.C.); lingyinzi1201@gmail.com (J.D.)

**Keywords:** benzovindiflupyr, zebrafish, gut microbiota, hepatic metabolomics, gut-liver axis

## Abstract

Benzovindiflupyr (BZF) is a newly developed succinate dehydrogenase inhibitor (SDHI) fungicide that is widely used in crop protection, but its potential effects on non-target aquatic organisms remain a concern. In this study, we exposed adult zebrafish (*Danio rerio*) to 5.0 and 50 μg/L BZF for 28 days. We investigated its impact on the gut–liver axis using a combination of microbiome, biochemical, histological, and metabolomic analyses. BZF exposure damaged intestinal structure, downregulated barrier-related genes, and altered the composition of the gut microbiota. At the same time, serum lipopolysaccharide (LPS) levels increased, which indicates impaired intestinal barrier integrity and microbial dysbiosis. In the liver, BZF caused histopathological alterations, increased serum ALT, AST, and ALP activities, enhanced oxidative stress, and upregulated inflammation-related genes. Liver metabolomic profiling further showed marked disturbances in redox balance and metabolic homeostasis. Correlation analysis also revealed significant associations between altered microbial taxa and differential liver metabolites. Taken together, these results suggest that BZF exposure disrupted intestinal homeostasis and was associated with hepatic metabolic disturbance in zebrafish, potentially through gut–liver axis perturbation. This study expands current understanding of the toxic effects of SDHI fungicides and provides useful evidence for the ecological risk assessment of BZF in aquatic environments.

## 1. Introduction

Succinate dehydrogenase inhibitors (SDHIs) are a relatively new class of fungicides that block electron transport from succinate to ubiquinone and thereby disrupt fungal energy metabolism [1]. Due to its broad-spectrum fungicidal activity and high efficacy, SDHI fungicides experienced a remarkable compound annual growth rate of 21.9% from 2004 to 2019 [2,3]. Benzovindiflupyr (BZF) is a pyrazole amide SDHI fungicide developed by Syngenta in 2012 and classified by the Fungicide Resistance Action Committee (FRAC) within the SDHI group (FRAC code 7) [4,5]. Since its introduction, BZF has been registered in many countries and is now widely applied to control a series of major crop diseases such as grey mold, cucumber anthracnose, and apple scab owing to its excellent fungicidal performance [6,7].

Although BZF provides agricultural benefits, its persistence in environment has raised increasing concern. Studies have reported that its half-life in soil ranges from 15 to 90 days. In addition, its low water solubility and limited photodegradation may allow it to remain in aquatic environments for longer time, which could increase its ecological risks to non-target organisms [8,9]. Residues of BZF have also been detected in agricultural products. For example, an Austrian survey conducted between 2019 and 2020 on 102 representative cow ration samples showed that 10% of the samples contained BZF, and 1% exceeded the EU default maximum residue limit of 10 µg/kg for animal feed [10].

Previous studies have shown that BZF significantly inhibits SDH activity and increases reactive oxygen species (ROS) production in zebrafish and earthworms [11,12]. Recent studies have also demonstrated that BZF stereoselectively affects locomotor behavior, metabolism and transcriptional regulation in *Xenopus laevis* tadpoles, which suggests that it may disrupt multiple physiological processes in non-target species [13]. In line with these findings, European Food Safety Authority (EFSA, 2015) classified BZF as highly toxic to aquatic organisms and noted its potential adverse effects on zebrafish embryo development [14]. However, despite evidence linking BZF exposure to SDH inhibition and ROS generation, its potential effects on intestinal barrier function, gut microbiota composition, hepatic metabolism, and gut–liver axis homeostasis in aquatic organisms remain insufficiently characterized. We therefore used zebrafish, a widely used vertebrate model with high genetic homology to humans and well-characterized physiological features, to investigate the toxic effects of BZF.

The gut–liver axis is a bidirectional communication network that links gut microbiota, intestinal barrier function and hepatic metabolism, and it plays a central role in maintaining metabolic homeostasis [15,16]. Increasing evidence indicates that gut microbial dysbiosis and intestinal barrier dysfunction can disturb this axis, promote the translocation of gut-derived inflammatory factors to the liver, and contribute to hepatic inflammation. Previous studies have shown that prothioconazole and triclosan can induce liver injury through gut microbiota-dependent mechanisms or gut–liver axis perturbation [17]. In addition, microbiota-modulated bile acids and gut-derived lipopolysaccharide (LPS) are important regulators of hepatic signaling and systemic metabolic homeostasis [18,19]. These findings suggest that gut–liver axis may represent an important target of environmental stressors.

However, the effects of BZF on intestinal barrier integrity, liver function and their interplay within the gut–liver axis are still unclear. To address this gap, adult zebrafish were exposed to BZF at 5.0 and 50 μg/L for 28 days. Because measured environmental concentrations of BZF in aquatic systems remain limited, the low-dose concentration was selected with reference to the chronic surface water exposure level estimated by the U.S. Environmental Protection Agency (EPA, 5.41 μg/L), and was therefore considered environmentally relevant. The higher concentration of 50 μg/L was set as a ten-fold higher toxicological model concentration to examine concentration-dependent responses and potential adverse effects under elevated exposure conditions. We then investigated whether BZF exposure was associated with gut–liver axis through gut microbial dysbiosis and intestinal barrier dysfunction, and whether these alterations were linked to hepatic injury.

## 2. Results

### 2.1. Effects of BZF on Growth Performance and Organ Indices

Body weight, body length, and *K* value remained unchanged following BZF exposure. In contrast, HSI showed a downward trend in the L-BZF group and was significantly reduced in the H-BZF group. ISI significantly decreased only in the H-BZF group (Figure S2).

### 2.2. BZF Exposure Disrupted Intestinal Structure and Barrier Integrity

**Figure 1:**
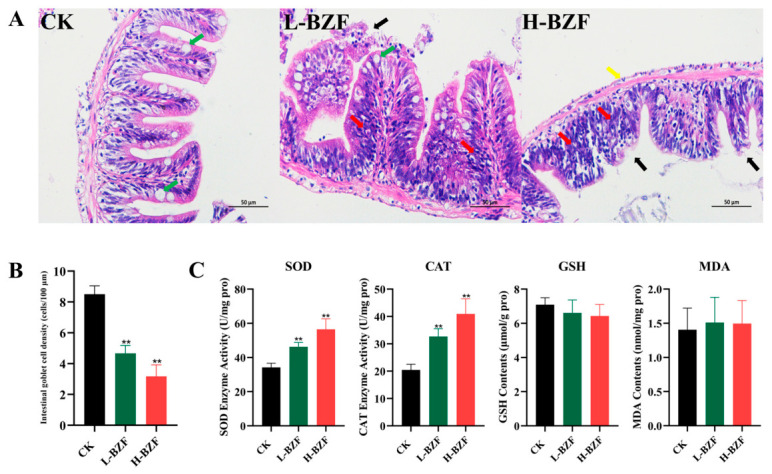
Intestinal histopathological analysis, goblet cell density and levels of intestinal oxidative stress markers in zebrafish intestines following BZF exposure. (**A**) Representative intestinal sections from each treatment group; green arrows denote goblet cells, black arrows highlight epithelial villus damage, red arrows show inflammatory infiltration, and yellow arrow points to thinning of intestinal wall. (**B**) Quantitative analysis of goblet cell density in intestinal tissues (*n* = 6); (**C**) Levels of intestinal oxidative stress markers, including SOD, CAT, GSH, and MDA (*n* = 6). ** *p* < 0.01 indicate significant differences compared with the CK group.

Histopathological analysis of intestinal tissues showed clear injury in BZF-exposed zebrafish, including intestinal epithelial villi damage, inflammatory infiltration, with more severe lesions observed in the H-BZF group. Intestinal wall thinning was also evident in the H-BZF group (Figure 1A). Goblet cell density decreased in a dose-dependent manner (Figure 1B), indicating impaired intestinal barrier integrity.

We also measured oxidative stress markers to assess intestinal physiological changes. Compared with the control group, SOD and CAT activities in both BZF treatment groups exhibited a dose-dependent increase. However, GSH and MDA levels did not change significantly (Figure 1C).

### 2.3. BZF Exposure Altered Gut Microbiota Composition

**Figure 2:**
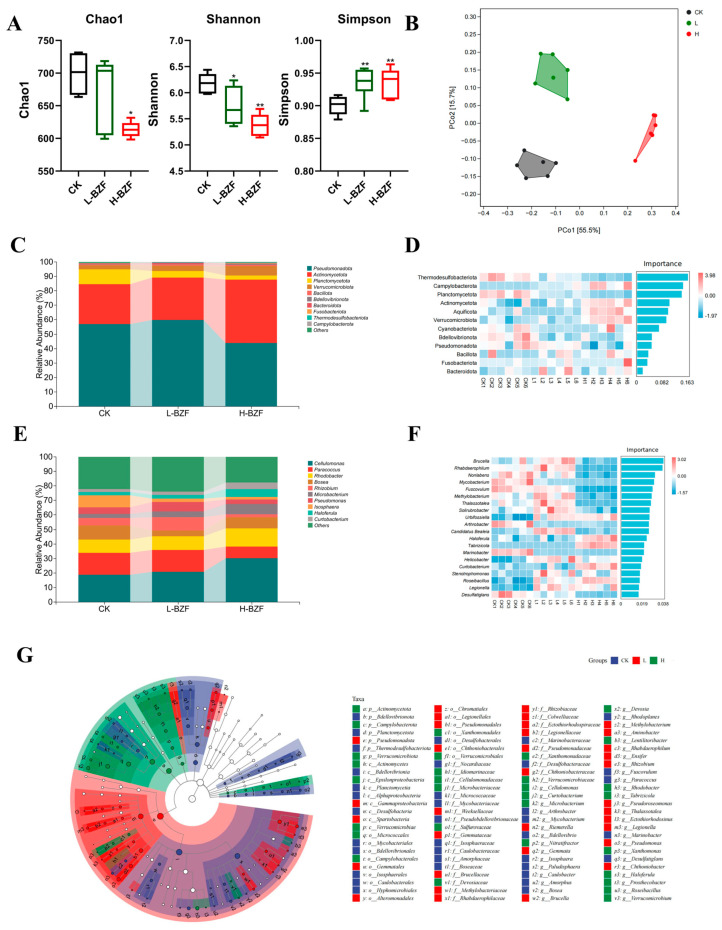
Gut microbiota profiles of zebrafish after 28−d BZF exposure. (**A**) Chao1, Shannon, and Simpson indices; (**B**) Bray–Curtis PCoA of microbial communities; (**C**) Taxonomic composition at the phylum level; (**D**) Random Forest classification based on phylum-level abundance data; (**E**) Taxonomic composition at the genus level; (**F**) Random Forest classification based on genus-level abundance data; (**G**) LEfSe analysis showing taxa enriched in different groups. For alpha-diversity indices, data are shown as boxplots (*n* = 6). * *p* < 0.05, ** *p* < 0.01.

We next analyzed the gut microbiota by 16S rRNA gene sequencing. Compared with the CK group, the H-BZF group showed a lower Chao1 index, both exposure groups showed lower Shannon values, and Simpson values increased in both BZF-treated groups (Figure 2A), indicating that BZF exposure altered gut microbial alpha diversity. PCoA using Bray–Curtis distances revealed clear separation among the CK, L-BZF, and H-BZF groups (Figure 2B), suggesting marked alterations in overall community structure.

We then examined changes in gut microbial composition in the CK and BZF-treated groups. Stacked bar charts show the microbial profiles at the phylum and genus levels (Figure 2C,E). Random Forest analysis was used to identify taxa contributing to group discrimination (Figure 2D,F). At the phylum level, *p_Pseudomonadota* predominated in the control group, whereas *p_Actinomycetota* and *p_Thermodesulfobacteriota* were enriched in the H-BZF group. Random Forest analysis identified *p_Thermodesulfobacteriota*, *p_Campylobacterota*, and *p_Planctomycetota* as major phyla contributing to group separation. At the genus level, *g_Cellulomonas* was enriched in the H-BZF group, while *g_Paracoccus* showed a decreasing trend. Moreover, *g_Brucella*, *g_Rhabdaerophilum*, and *g_Nonlabens* were identified as important discriminatory genera. LEfSe analysis showed that different taxa were enriched in different groups (Figure 2G), consistent with compositional shifts in the gut microbiota after BZF exposure.

### 2.4. BZF Exposure Was Associated with Hepatic Alterations in Zebrafish

**Figure 3:**
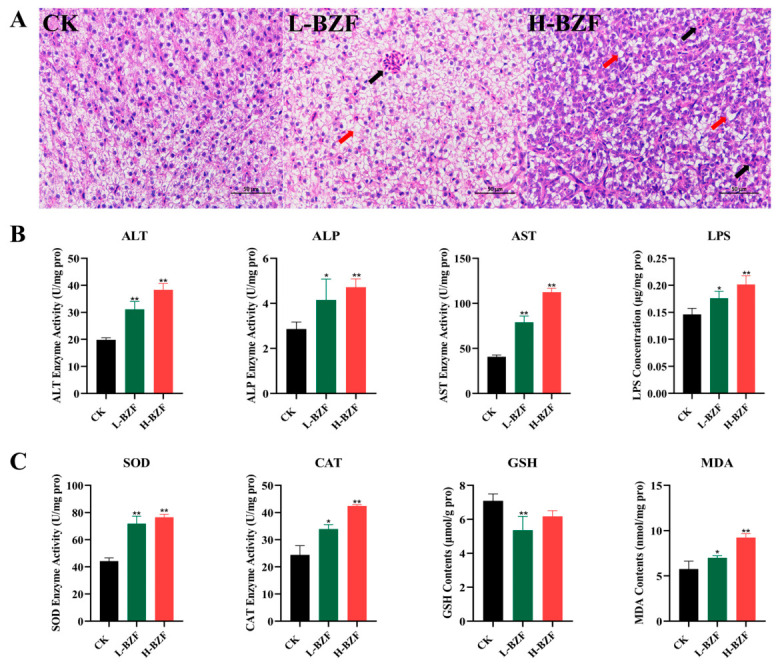
Liver histopathological analysis and biochemical indicators of liver injury, inflammation and oxidative stress in zebrafish after BZF exposure. (**A**) Representative liver histopathological sections from each treatment group; red arrows indicate hepatocellular swelling, black arrows indicate inflammatory infiltration; (**B**) Serum biochemical indicators related to hepatic and gut-liver axis (ALT, AST, ALP and LPS); (**C**) Hepatic SOD, CAT activities, GSH content and MDA concentration. Data are presented as mean ± SD (*n* = 6) * Indicates significant differences compared with CK group; * *p* < 0.05 and ** *p* < 0.01 indicate significant differences compared with the CK group.

Histopathological analysis revealed alterations in the liver of BZF-exposed zebrafish, including hepatocellular swelling, inflammatory infiltration and disordered hepatic cord structure, with more severe lesions in the H-BZF group (Figure 3A). Serum ALT, AST, ALP activities and LPS level were also increased after BZF exposure (Figure 3B). Hepatic SOD and CAT activities also increased significantly in both BZF-treated groups, whereas GSH content decreased significantly only in the L-BZF group. MDA content was significantly elevated in both exposure groups, especially in the H-BZF group (Figure 3C). Collectively, these histopathological and biochemical observations suggest that BZF exposure was associated with hepatic alterations in zebrafish, with more pronounced effects at the higher exposure concentration.

### 2.5. BZF Exposure Induced Hepatic Metabolic Disturbance

**Figure 4:**
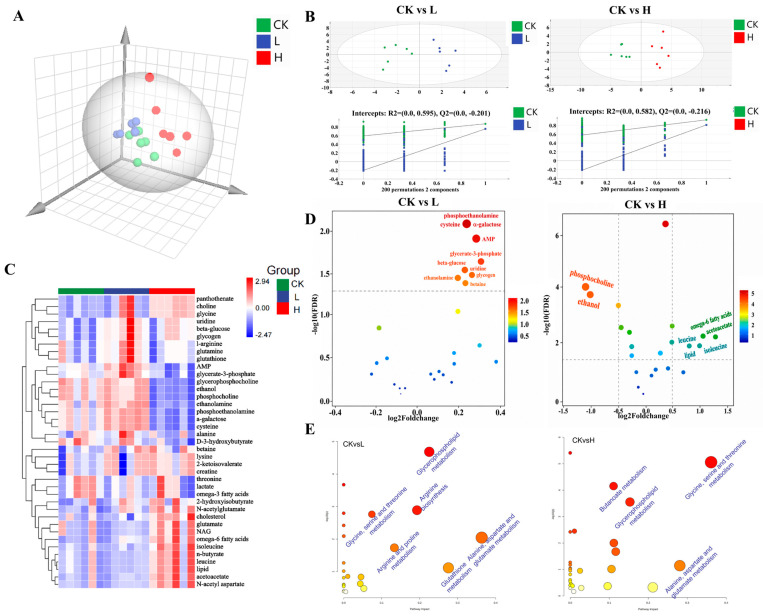
Hepatic metabolomic profiling of zebrafish following BZF exposure. (**A**) PCA score plot showing global metabolic variation among groups; (**B**) PLS-DA score plots and permutation tests for CK vs. L-BZF and CK vs. H-BZF comparisons; (**C**) Heatmap of differential metabolites across groups; (**D**) Volcano plots of significantly altered metabolites in the L-BZF (left) and H-BZF (right) groups compared with CK group; (**E**) KEGG pathway enrichment analysis of differential metabolites.

To examine metabolic changes in the liver after BZF exposure, we performed ^1^H NMR-based metabolomic analysis. A 600 MHz ^1^H NMR spectrum of liver tissue is shown in Figure S3. Principal component analysis (PCA) showed a separation trend between CK and two BZF-treated groups (Figure 4A), suggesting that BZF affected the overall hepatic metabolic profile. Partial least squares discriminant analysis (PLS-DA) provided clearer discrimination among groups, and permutation testing confirmed the validity of the model (Figure 4B).

Based on the PLS-DA model, 39 differential metabolites were identified using a variable importance in projection threshold (VIP > 1) together with a false discovery rate (FDR) < 0.05. Heatmap and volcano plot analyses showed significant changes in metabolite abundance after BZF exposure (Figure 4C,D). In the L-BZF group, 27 metabolites were significantly altered, mainly involving amino acids, choline-containing metabolites, carbohydrates, glutathione-related metabolites and energy metabolism intermediates. In the H-BZF group, 30 metabolites were significantly altered, with broader changes in amino acid metabolism, lipid-related metabolites, short-chain fatty acid and ketone body-related metabolites, and acetylated compounds.

We then performed KEGG pathway enrichment analysis to examine the biological significance of the altered metabolites (Figure 4E). Glycine-serine-threonine metabolism pathway, alanine-aspartate-glutamate metabolism and glycerophospholipid metabolism pathway were significantly enriched in both BZF-treated groups. In addition, butanoate metabolism was particularly enriched in the H-BZF group, while arginine and proline metabolism, glutathione metabolism and arginine biosynthesis were more prominently affected in the L-BZF group. These metabolomic data suggest that BZF exposure altered several liver pathways, especially those related to amino acid, glycerophospholipid metabolism and redox regulation, which is consistent with the hepatic injury observed in zebrafish.

### 2.6. BZF Exposure Altered Gene Expression in Liver and Intestine

**Figure 5:**
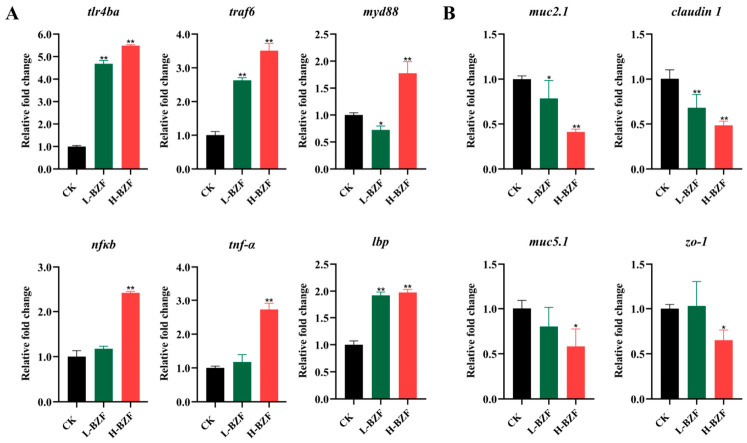
Relative fold changes of gene expression in zebrafish. (**A**) Hepatic expression levels of genes associated with the gut–liver axis, inflammatory signaling and LPS binding protein; (**B**) Intestinal expression levels of genes involved in intestinal barrier integrity. Data are presented as mean ± SD (*n* = 6). * *p* < 0.05 and ** *p* < 0.01 indicate significant differences compared with the CK group.

qRT-PCR analysis showed that BZF exposure significantly altered the expression of genes related to hepatic inflammatory signaling and intestinal barrier integrity. In the liver, *tlr4ba*, *traf6* and *lbp* were significantly upregulated in both BZF-treated groups; while *nfκb* and *tnf-α* increased significantly only in the H-BZF group. Interestingly, *myd88* showed a biphasic pattern with downregulation in the L-BZF group and upregulation in the H-BZF group (Figure 5A). In the intestine, both exposure groups exhibited significant decreases in *muc2.1* and *claudin 1*, whereas significant reductions in *muc5.1* and *zo-1* were detected only in the H-BZF group (Figure 5B). Together, these alterations indicate that BZF exposure was associated with altered expression of inflammation-related genes in the liver and impaired intestinal barrier-related gene expression.

### 2.7. Correlation Between Gut Microbiota and Hepatic Metabolic Profiles

**Figure 6:**
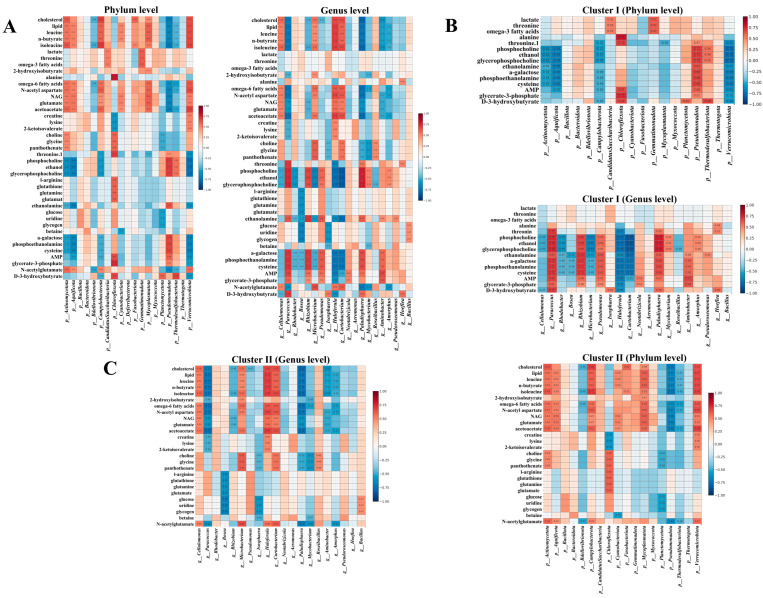
Correlation analysis between altered gut microbiota (phylum and genus levels) and significantly changed metabolites. (**A**) Pearson correlation heatmap illustrating the associations between altered gut microbial taxa (phylum and genus levels) and significantly changed liver metabolites; (**B**) Cluster I: group of metabolites identified through hierarchical clustering exhibiting similar correlation patterns with gut microbiota; (**C**) Cluster II: distinct group of metabolites with contrasting correlation profiles.

We then calculated Pearson correlations between the taxa altered by BZF exposure and the differential hepatic metabolites. The resulting correlations were visualized using heatmaps of Pearson correlation coefficients (Figure 6). At the phylum level, several microbial phyla were significantly correlated with specific liver metabolites. In particular, 25, 21, and 21 metabolites were significantly correlated with *p_Verrucomicrobiota*, *p_Pseudomonadota*, and *p_Actinomycetota*, respectively (Figure 6A). Other phyla, such as *p_Campylobacterota* are also significantly correlated with the metabolite subset. These results indicate broad and specific associations between microbial composition and host metabolism at the phylum level. Correspondingly, a similar pattern was also observed at the genus level (Figure 6A), where several genera, such as *g_Paracoccus*, *g_Haloferula* and *g_Paludisphaera* showed significant correlations with a large number of liver metabolites. It is worth noting that *g_Paracoccus* was associated with 26 altered metabolites. Hierarchical clustering analysis further revealed two major metabolite clusters based on their correlation profiles with gut microbiota (Figure 6B,C). These correlation patterns suggest a close association between gut microbial dysbiosis and hepatic metabolic disturbance in BZF-exposed zebrafish.

## 3. Discussion

Benzovindiflupyr is a novel SDHI fungicide that has been increasingly used in crop protection. Although BZF is highly effective against plant pathogens, its potential ecological risks have attracted increasing attention, particularly because of its environmental persistence and possible toxicity to non-target aquatic organisms. Previous studies have reported hepatocellular alterations and colonic mucosal hyperplasia in mammals exposed to high doses of BZF [20,21,22]. However, its toxic effects, especially on intestinal barrier integrity and gut–liver axis homeostasis in aquatic species remain insufficiently characterized.

Intestinal injury was one of the prominent responses observed after BZF exposure. Although general growth-related parameters were not markedly affected, a reduction in ISI in the H-BZF group suggested impaired intestinal status [23,24,25]. Histopathological analysis further revealed villi damage, inflammatory infiltration, and intestinal wall thinning, indicating structural damage to the intestinal epithelium [26]. Moreover, goblet cell density decreased after BZF exposure. Goblet cells can secrete Muc2, which is a key component of intestinal mucus layer and can protect intestine from microbial invasion and chemical damage [26,27]. Therefore, these reductions may weaken mucosal defense and increase inflammatory sensitivity [28,29]. At the molecular level, the downregulation of *muc2.1*, *muc5.1*, *zo-1*, and *claudin 1* further supports the view that BZF impaired intestinal barrier integrity, especially in the H-BZF group [30,31]. The elevated intestinal SOD and CAT activities also suggest activation of antioxidant defense responses against BZF-induced stress [32]. Taken together, these results suggest that BZF exposure disrupted intestinal homeostasis and compromised barrier function in zebrafish.

Gut microbiota dysbiosis was another important response to BZF exposure. The decrease in alpha diversity and the clear separation in beta diversity indicates a marked restructuring of the gut microbial community [33,34,35]. At the phylum level, the shift from *p_Pseudomonadota* to enriched *p_Actinomycetota* and *p_Thermodesulfobacteriota* suggests a shift toward a pro-inflammatory intestinal profile [36,37]. This microbial dysbiosis was closely linked to hepatic metabolic disturbance via the gut–liver axis, as evidenced by the significant correlations between *p_Actinomycetota* (associated with 21 altered liver metabolites) and hepatic profiles. At the genus level, the marked increase in *g_Cellulomonas*-a potential biomarker for chemical-induced stress and the significant decline in *g_Paracoccus* (correlated with 26 altered liver metabolites) further highlight the functional impact of BZF on host homeostasis [38,39]. These results indicate that the altered gut microbiota was not only structurally disrupted, but also functionally connected to the reprogramming of hepatic metabolism under BZF exposure. Similar observations have also been reported for other SDHI fungicides such as boscalid, in which gut microbiota dysbiosis was closely associated with hepatic oxidative stress and lipid metabolic reprogramming [40,41].

In line with the disruption of intestinal homeostasis, hepatic alterations were observed in BZF-exposed zebrafish. HSI showed a downward trend after BZF exposure, particularly in the H-BZF group, suggesting a potential alteration in hepatic status [42]. This interpretation was supported by histopathological lesions and increased serum ALT and AST activities, which provide complementary evidence for hepatic responses in BZF-exposed zebrafish. Serum ALP activity was also elevated after BZF exposure; however, this change should be interpreted cautiously because ALP is not liver-specific. Moreover, intestinal ALP has been reported to participate in LPS detoxification and intestinal inflammatory regulation in zebrafish. Therefore, considering the intestinal barrier damage and increased serum LPS observed in this study, ALP elevation may reflect a broader gut–liver axis-related response rather than liver-specific injury alone [33,43]. In addition, increased SOD and CAT activities, elevated MDA levels, and decreased GSH levels suggest that oxidative stress and redox imbalance may be involved in the hepatic alterations associated with BZF exposure [44]. Together, these results suggest that BZF exposure was associated with hepatic responses in zebrafish, with more pronounced effects at the higher exposure concentration.

To further characterize hepatic metabolic alterations associated with gut–liver axis perturbation, we analyzed hepatic metabolomic profiles, given the close relationship between gut microbiota and hepatic function. BZF exposure markedly perturbed alanine, aspartate and glutamate metabolism, glycine, serine and threonine metabolism, glycerophospholipid metabolism, and glutathione metabolism. These pathways are closely linked to redox balance, energy production, membrane integrity, and detoxification processes [45]. Additionally, perturbations in butanoate metabolism and other SCFA-related pathways were also observed, which may reflect metabolic alterations associated with gut microbial dysbiosis [46].

At the molecular level, our results suggest that BZF exposure was associated with activation of hepatic inflammation-related signaling. The elevated serum LPS level, together with impaired intestinal barrier integrity, indicates enhanced translocation of gut-derived inflammatory stimuli into the circulation [47]. LBP can bind circulating LPS and facilitate its recognition by hepatic immune signaling components, thereby promoting downstream inflammatory responses [48]. In the present study, the upregulation of *lbp*, *tlr4ba*, and *traf6* may reflect changes in inflammation-related responses following BZF exposure [49]. Moreover, the increased expression of *myd88*, *nfκb*, and *tnf-α*, particularly in the H-BZF group, was consistent with enhanced hepatic inflammatory signaling [50]. However, unlike the canonical mammalian LPS–TLR4 pathway, zebrafish Tlr4 does not appear to function as a typical LPS receptor, and LPS responses in fish have been reported to occur through Tlr4- and MyD88-independent mechanisms [51,52]. Therefore, the increased expression of tlr4ba, myd88, nfκb, and tnf-α in this study should be regarded as evidence of altered inflammation-related gene expression, rather than direct proof of canonical LPS–TLR4 pathway activation. Further protein-level and functional validation will be needed to clarify the exact role of this pathway in BZF-associated hepatic responses. Together with the disturbances observed in hepatic amino acid and lipid metabolism, these results support the view that gut–liver axis perturbation may contribute to BZF-induced hepatotoxicity. Consistent with our findings, recent studies have highlighted that other SDHI fungicides like boscalid can induce gut microbiota dysbiosis and impair intestinal metabolic homeostasis, which triggers hepatic oxidative stress and lipid metabolism disorders in zebrafish through gut–liver axis [40,41]. Nevertheless, because these observations are based on transcript levels only, additional protein-level or functional validation would be needed to confirm pathway activation.

## 4. Materials and Methods

### 4.1. Zebrafish Maintenance and Chemicals

AB wild-type adult zebrafish (4 months old) were obtained from Hongda Gaofeng Aquarium (Beijing, China). Before exposure, we acclimated the fish for two weeks in aerated, dechlorinated tap water (pH 7.2 ± 0.2, conductivity 300 ± 20 μS/cm) at 27 ± 1 °C, with a 14 h:10 h light/dark photoperiod and fed them twice daily. The water was renewed every day. Alta Scientific Co., Ltd. (Tianjin, China) supplied benzovindiflupyr (BZF) (CAS: 1072957-71-1; purity 99.9%). We prepared the stock solution in analytical-grade acetone and stored it at 4 °C. A full list of chemicals and reagents is provided in Table S1.

All animal procedures received approval from the Independent Animal Ethics Committee of China Agricultural University and adhered to established animal welfare guidelines (protocol number: AW61705205-6-01).

### 4.2. Exposure Experiments

After acclimation, we randomly assigned adult zebrafish to 20 L glass tanks containing 10 L of exposure solution and exposed them for 28 days. Three groups were included: a solvent control group (CK) and two BZF exposure groups containing 5.0 μg/L (L-BZF) and 50 μg/L (H-BZF) BZF. The final concentration of acetone was 0.001% (*v/v*) in all groups. Each group had six replicate tanks, with 15 fish per tank (90 fish per group) and we maintained an equal proportion of males and females in each group. The experimental design is presented in Figure S1.

With reference to the modeled EPA surface-water exposure estimate, we selected 5.0 μg/L as a low-dose exposure, and 50 μg/L as a higher concentration to further investigate the potential toxicological mechanisms of BZF. To maintain stable exposure levels, we renewed the test solutions daily and added fresh BZF stock solution after each water renewal. As previously described [53,54,55], the actual exposure concentrations in each group were determined using an HPLC-MS/MS system comprising a Thermo Fisher Scientific Ultimate 3000 and a TSQ Quantum Access MAX spectrometer. Detailed analytical conditions and concentration data are summarized in Appendix S1 and Tables S2 and S3. Fish mortality remained below 1.5%, and detailed data are presented in Table S4.

### 4.3. Sample Collection

After 28 days of exposure, we anesthetized the zebrafish on ice and recorded total body weight and total body length before dissection. The liver and intestine were removed, weighed and collected for subsequent analyses. We calculated Fulton’s condition factor (K), Hepatosomatic Index (HSI), and Intestine somatic Index (ISI) using the following equations. Tissue samples were immediately frozen in liquid nitrogen and stored at −80 °C until analysis.

K = Total body weight (g) × 100/ Total body length (cm)^3^

HSI (%) = Liver weight (g) × 100/ Total body weight (g)

ISI (%) = Intestine weight (g) × 100/ Total body weight (g)

### 4.4. Histopathological Examination

For histopathological examination, one fish was randomly selected from each replicate tank, resulting in six fish per group (*n* = 6). Histopathological examination and goblet cell quantification were performed using standard procedures detailed in Appendix S2.

### 4.5. Biochemical Analysis and Determination of Oxidative Stress

For serum biochemical analysis, serum samples from four fish within the same replicate tank were pooled to generate one biological replicate, resulting in six replicates per group (*n* = 6). Liver and intestinal tissues from the same four fish in each replicate tank were pooled separately for oxidative stress analysis, resulting in six tank-level biological replicates per group (*n* = 6). Serum ALT, AST and ALP activities were quantified by commercial assay kits (Jiancheng, Nanjing, China). Serum LPS concentration was determined using an ELISA kit (Enzyme Link Biotechnology, Shanghai, China). Oxidative stress was evaluated by measuring superoxide dismutase (SOD) and catalase (CAT) activities, as well as glutathione (GSH) and malondialdehyde (MDA) levels, using commercial kits (Jiancheng, Nanjing, China). The total protein concentration was determined with a BCA protein assay kit (Jiancheng, Nanjing, China) to normalize enzyme activities and MDA levels.

### 4.6. Microbiome Analysis of Gut Contents

Gut contents from three fish randomly selected from each replicate tank were pooled as one biological sample, and six pooled samples were prepared for each group (*n* = 6). DNA extraction, 16S rRNA gene amplification, sequencing, and bioinformatic analysis were performed as described in Appendix S3.

### 4.7. Hepatic Metabolomic Analysis

We performed untargeted liver metabolomics using ^1^H NMR spectroscopy. Livers from three zebrafish randomly selected from each replicate tank were pooled as one biological sample, and six biological replicates were prepared for each group (*n* = 6). The detailed sample preparation procedure and NMR parameters are provided in Appendix S4. Metabolic pathway enrichment analysis was performed in MetaboAnalyst (v6.0) based on the KEGG database.

### 4.8. Gene Expression Analysis

We analyzed gene expression to evaluate inflammatory responses in the liver and barrier-related changes in the intestine. Three fish were randomly selected from each replicate tank. Liver and intestinal tissues were collected from the same fish and pooled separately as biological replicates for each tissue, resulting in six biological replicates per group (*n* = 6). Total RNA extraction, cDNA synthesis, and qRT-PCR procedures followed protocols described previously [56]. A list of primers (Sangon Biotech, Shanghai, China) is provided in Table S5. Detailed procedures are found in Appendix S5.

### 4.9. Statistical Analysis

Data are expressed as mean ± SD. Normality and homogeneity of variance were assessed by the Shapiro–Wilk and Levene’s tests. Group comparisons utilized one-way ANOVA followed by Tukey’s post hoc test using SPSS 25.0 (IBM, USA). When the data did not meet the assumptions for parametric analysis, the non-parametric Kruskal–Wallis test followed by Dunn’s post hoc test was used. For microbiome analyses, beta-diversity differences were evaluated using Bray–Curtis distances and tested with PERMANOVA (999 permutations). Random Forest and LEfSe analyses were used to identify taxa contributing to group separation. Pearson correlation coefficients were calculated in R software (Version 4.5.1) and visualized using heatmaps. Figures were generated in GraphPad Prism 8.0 (San Diego, CA, USA). A *p*-value < 0.05 was considered statistically significant.

## 5. Conclusions

In summary, BZF exposure induced gut microbial dysbiosis, intestinal structure damage, and impaired intestinal barrier integrity in zebrafish. These changes were accompanied by elevated serum LPS levels and altered expression of inflammation-related genes in the liver, suggesting a potential association between gut–liver axis-related responses and hepatic alterations following BZF exposure. Liver metabolomics further showed that amino acid and lipid metabolism were disordered, indicating disrupted metabolic homeostasis. Notably, these effects were more pronounced in the H-BZF group. Collectively, these findings suggest that gut–liver axis-related changes are associated with BZF exposure. While this study provides new insights into the potential toxicological effects of SDHI fungicides, further investigations are required to validate these associations and elucidate the underlying mechanisms, including functional verification of intestinal barrier integrity and the role of gut-derived inflammatory signals in hepatic responses.

## Data Availability

The raw data supporting the conclusions of this article will be made available by the authors on request.

## Supplementary Materials

The following supporting information can be downloaded at: https://www.mdpi.com/article/10.3390/ijms27125455/s1.
