# Supplementary material for: Benzovindiflupyr Is Associated with Metabolic Homeostasis Disturbance and Gut–Liver Axis Alterations in Zebrafish: Insights from a Multi-Omics Approach
Source: Int J Mol Sci. 2026 Jun 17;27(12):5455. doi: 10.3390/ijms27125455 (PMC13299776; doi:10.3390/ijms27125455)
Supplement: Supplementary file 1 [file ijms-27-05455-s001.zip › ijms-4338012-supplementary.pdf]

Supporting information

Benzovindiflupyr is associated with metabolic homeostasis disturbance and gut-liver axis alterations in zebrafish: insights from a multi-omics approach

Jiyan Miao[a], Shihang Han[a], Xinrui Dang[a], Qi Chen[a], Jinling Diao[a], Wentao Zhu[a]*

[a] Innovation Center of Pesticide Research, Department of Applied Chemistry, College of Science, China Agricultural University, Beijing 100193, China.

Corresponding author:

*Wentao Zhu: Innovation Center of Pesticide Research, Department of Applied Chemistry, College of Science, China Agricultural University, Beijing 100193, China.

E-mail address: wentaozhu@cau.edu.cn.

Number of Appendices: 5

Number of Tables: 5

Number of Figures: 3

Table of contents

Appendix S1. The instrument parameters of LC-MS/MS.

Appendix S2. Detailed procedures for histopathological examination and goblet cell quantification.

Appendix S3. Detailed procedures for gut microbiome analysis by 16S rRNA gene sequencing.

Appendix S4. Detailed methods for hepatic metabolomic analysis and NMR parameters.

Appendix S5. RNA isolation and mRNA expression analysis.

Table S1. The reagents and chemicals used in this study.

Table S2. Chromatography and mass spectrometry information of benzovindiflupyr.

Table S3. Measured background and working concentrations of BZF by experimental group.

Table S4. The mortality of zebrafish during the exposure recorded for each experimental group.

Table S5. Information of primers in qRT-PCR.

Figure S1. *Z*ebrafish experimental design.

Figure S2. Body weight, body length, K, HSI and ISI values of zebrafish in different groups after BZF exposure.

Figure S3. Representative 600MHz ^{1}H NMR spectra of zebrafish liver samples.

Appendix S1. The instrument parameters of LC-MS/MS.

Chromatographic separation was performed using an Eclipse C18 column (2.1 mm × 100 mm, 1.7 μm). The mobile phase consisted of acetonitrile and 0.1% formic acid in water (90:10, v/v). Selective reaction monitoring (SRM) in negative ion mode was employed for both qualitative and quantitative analysis. The column temperature was maintained at 30 °C, and the total run time was 9.5 minutes. The ion source temperature was set to 350 °C, with a nebulizer pressure of 45 psi and an electrospray voltage of 3500 V. The precursor and corresponding product ions used for SRM detection of benzovindiflupyr are listed in Table S2.

Appendix S2. Detailed procedures for histopathological examination and goblet cell quantification.

Liver and intestinal tissues were collected from six fish per group (n = 6). After fixation in 10% formalin for 24 hours, tissues were dehydrated in ethanol and were cleared with xylene. They were then embedded in paraffin, cut into 5 μm sections, stained with hematoxylin and eosin (H&E) and examined under an Olympus BX51 microscope (Olympus, Japan). Goblet cell density was used as an indicator of intestinal barrier status and was calculated as the number of goblet cells per unit area. Histological parameters were analyzed using ImageJ software (NIH, USA).

Appendix S3. Detailed procedures for gut microbiome analysis by 16S rRNA gene sequencing.

After 28 days of exposure, zebrafish were fasted for 24 h before sampling. Gut contents from six zebrafish were pooled as one biological sample, and six pooled samples were prepared for each group (n = 6). Genomic DNA was extracted using the QIAamp DNA Stool Mini Kit (Qiagen, Germany) in accordance with the provided instructions. DNA integrity and concentration were assessed by 1% agarose gel electrophoresis and UV spectrophotometry, respectively. The V3–V4 region of the bacterial 16S rRNA gene were amplified with primers 338F and 806R and sequenced on the Illumina MiSeq platform (2×300 bp paired-end). Sequence data were processed using QIIME2 (version 2019.4), followed by analysis with Vsearch and the SILVA 138 database.

Appendix S4. Detailed methods for hepatic metabolomic analysis and NMR parameters.

Detailed methods for hepatic metabolomic analysis:

Approximately 50 ± 1 mg of each pooled sample was used for metabolite extraction. Each sample was extracted twice by homogenization (3 minutes) in pre-chilled methanol/water mixture (2:1; v/v) with an MM 400 homogenizer (Retsch GmbH, Germany). After centrifugation at 12,000 rpm for 10 min at 4 °C, the supernatants from the two extractions of the same sample were combined, evaporated under nitrogen, and reconstituted in 550 μL phosphate buffer. The buffer solution was prepared with deuterium oxide (D_2O) and contained 1 mM sodium trimethylsilylpropionate (TSP) for chemical shift referencing. After centrifugation at 12,000 rpm for 10 min at 4 °C, the supernatant was aliquoted to 5 mm NMR tubes.

NMR parameters:

^{1}H NMR spectra of liver samples were acquired using a Bruker AVANCE III HD 600 MHz spectrometer equipped with an inverse cryogenic probe, operating at 300 K. Water suppression was applied during spectral acquisition. To minimize macromolecular signal interference and retain low-molecular-weight metabolites, the Carr–Purcell–Meiboom–Gill (CPMG) spin-echo pulse sequence (recycle delay–90°–(τ–180°–τ)_n–acquisition) was employed with a spin–spin relaxation delay of 80 ms. The spectral width was set to 20 ppm, with 32k data points. A total of 64 transients were collected for each sample, with a relaxation delay of 4 s and an acquisition time

of 2.72 s.

Multivariate statistical analysis was performed using SIMCA-P software (version 14.1, Umetrics, Sweden). The binned NMR data were imported into SIMCA-P for analysis. Principal component analysis (PCA) was first applied to reveal intrinsic clustering patterns and detect outliers. Partial least squares discriminant analysis (PLS-DA) was then conducted to identify group differences. Metabolite assignments were performed based on ^1H NMR chemical shifts, peak multiplicity, and comparison with public NMR spectral databases and published literature. Because authentic reference standards and spiking experiments were not used for further confirmation, the identified metabolites were considered putatively annotated metabolites rather than fully confirmed identifications. Variable Importance in Projection (VIP) scores from the PLS-DA loading plots were used to screen discriminating metabolites. Statistical significance of these metabolites was further assessed using SPSS 19.0 (IBM, USA) with one-way ANOVA or Kruskal–Wallis tests, depending on data distribution. Metabolites with VIP > 1 and FDR-adjusted $p < 0.05$ were considered differential metabolites.

Appendix S5. RNA isolation and mRNA expression analysis.

Total RNA was extracted using TRIzol reagent (Tiangen, Beijing, China) following the manufacturer's instructions. Briefly, 550 μL of reagent was added to homogenize the tissue, followed by chloroform extraction, isopropanol precipitation, 75% ethanol wash, and dissolution in DNase/RNase-Free H_2O. RNA concentration and purity were assessed using a spectrophotometer (DS-11, DeNovix, USA). Subsequently, 1500 ng of total RNA was reverse transcribed into cDNA using a Fast Quant RT Kit (Tiangen Biotech, Beijing, China). Real-time quantitative PCR (RT-qPCR) was performed with SuperReal PreMix Plus (SYBR Green) in a Bio-Rad CFX 96 PCR system (Bio-Rad, USA). The PCR amplification program consisted of an initial denaturation at 95 °C for 60 s, followed by 40 cycles of 95 °C for 15 s and 60 °C for 60 s. Melting curve analysis was performed to confirm primer specificity. Primers were designed using the NCBI Primer-BLAST tool (https://www.ncbi.nlm.nih.gov/tools/primer-blast/), synthesized by Sangon Biotech (Shanghai, China), and their sequences are listed in Table S5. Gene expression levels were normalized to the housekeeping gene *β-actin*, and relative mRNA expression was calculated using the $2^{-\Delta\Delta Ct}$ method.

Table S1. The reagents and chemicals used in this study.

Reagent or chemical	Abbreviation	Purity	CAS number	Manufacturer
Benzovindiflupyr	BZF	99.9 %	1072957-71-1	Alta Scientific Co., Ltd. (Tianjin, China)
Methanol	--	HPLC-grade	67-56-1	ANPEL Laboratory Technologies Inc (Shanghai, China)
Acetonitrile	--	HPLC-grade	75-05-8	ANPEL Laboratory Technologies Inc (Shanghai, China)
Acetone	--	Analytical Reagent (AR) grade	67-64-1	Beijing Institute of Chemical Reagents Co., Ltd.
Formic acid	--	HPLC-grade	64-18-6	ANPEL Laboratory Technologies Inc (Shanghai, China)
Water	H_2O	ultra-pure	7732-18-5	Milli-Q (Merck KGaA, Darmstadt, Germany)

Table S2. Chromatography and mass spectrometry information of BZF.

**Compound**	**t_R (min)**	**Molecular Weight (g/mol)**	**Parent ion (m/z)**	**Product ion (m/z)**	**Fragmentor (V)**	**Collision energy (eV)**
Benzovindiflupyr	8.05	398.23	395.9	368.29	80	15
				90.98	80	30

t_R: Retention time

Table S3. Measured background and working concentrations of BZF by experimental group (n = 6 each). Data are presented as mean ± SD. ND, not detected

Experimental groups (nominal concentration of BZF)	Detected concentrations of BZF in each experimental group
Control (0)	ND
L-BZF group (5 μg/L)	5006.25 ± 5.66 ng/L
H-BZF group (50 μg/L)	50034.17 ± 9.73 ng/L

The sensitivity of the method was evaluated using the Method Detection Limit (MDL) Limit of Detection (LOD) and Limit of Quantification (LOQ) . MDL is calculated by determining the BZF concentration that can be reliably detected with a specified level of confidence, typically using replicate blank samples at a signal-to-noise ratio (S/N) of 3. LOD is calculated by determining the BZF concentration at a S/N of 3. LOQ is calculated by determining the BZF concentration at a S/N of 10.

The MDL, LOD and LOQ for BZF are as follows:

MDL: 0.157 μg/L; LOD: 0.112 μg/L; LOQ: 0.373 μg/L

Table S4. The mortality of zebrafish during the exposure recorded for each experimental group.

Group	Number of casualty	Mortality rate (%)
CK	1	0.67
L-BZF	0	0
H-BZF	2	1.11

Table S5. Information of primers in qRT-PCR

Gene	Sequences of primers	Amplicon Length	Accession Number (bp)
β-actin	Forward: GGTTTTGCTGGAGATGATGC Reverse: CACAATACCGTGCTCAATGG	NM_131031.2	171
tlr4ba	Forward: TGCGATGAAAGTAAGATCCAC Reverse: ACAGGCAAGCAGACTTGGT	NM_0011305 41.2	128
myd88	Forward: AACAACTTCGCTGGATAA Reverse: GTTACTGGAATCGCCTCA	NM_212814.2	82
traf6	Forward: CCAATTCGGTTACTGCTTAA Reverse: CCTGATGGATTTCCTAATG	NM_0010452 33.1	139
nfκb	Forward: AACGAGGCTGAGAGGAGTCT Reverse: ATCAGCCGAACCACACTGAG	NM_0013538 73.1	80
tnf-α	Forward: ACAAGATGGAAGTGTGCTGAGA Reverse: ATTTCAAGCCACCTGAAGAAAA	NM_212859.2	164
muc2.1	Forward: CATCAATGTGGCTCCTGA Reverse: CCTTTCTTTCACATCCAGGA	NM_0011105 15	123
muc5.1	Forward: TGAGATGCTATTTGTGCCAAC Reverse: TGGTTCTATTCACGTGTCTCT	NM_0010403 33	120
zo-1	Forward:	NM_212686	173

	CCCGATGAGGATGAAGAA<br>Reverse:<br>TGTGCTGGTTGAGTTTGG		
claudin 1	Forward:<br>TGGAGCATCGCTGTATCTG<br>Reverse:<br>ACGACTTTGCGGAGGGTA	NM_131764.1	115
lbp	Forward:<br>CACTTCGCCATTGTTCTG<br>Reverse:<br>CCAATTCGGTTACTGCTTAA	NM_0010452<br>62	125

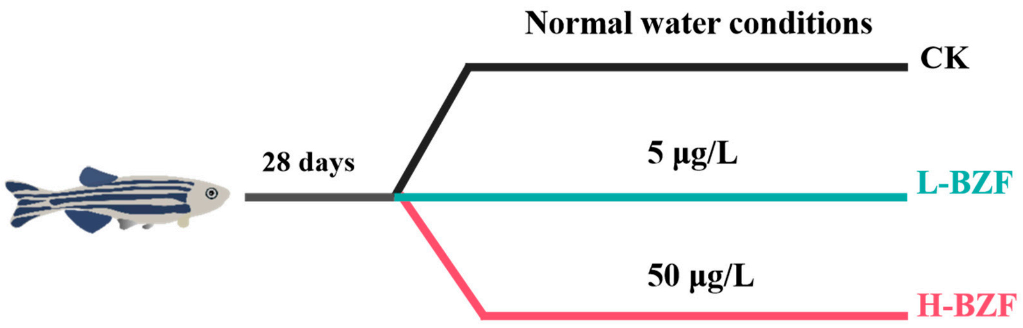

Figure S1. Zebrafish experimental design. Fish were exposed to benzovindiflupyr (BZF) through water at concentrations of 5 µg/L (L-BZF) and 50 µg/L (H-BZF) for 28 days. Fish body length was measured as total length (TL), defined as the distance from the tip of the snout to the end of the tail fin. CK: Control group; L-BZF: (low-dose group, 5.0 µg/L) and H-BZF: (high-dose group, 50 µg/L).

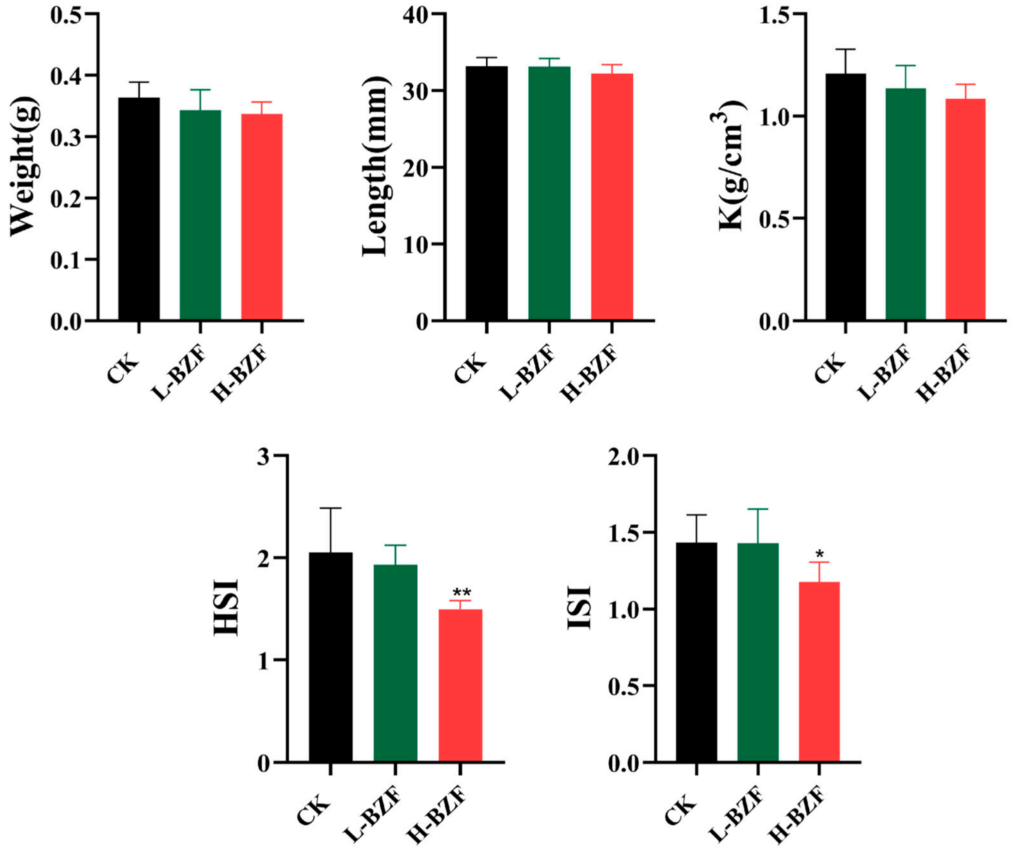

Figure S2. Body weight, body length, K, HSI and ISI values of zebrafish in different groups after BZF exposure.

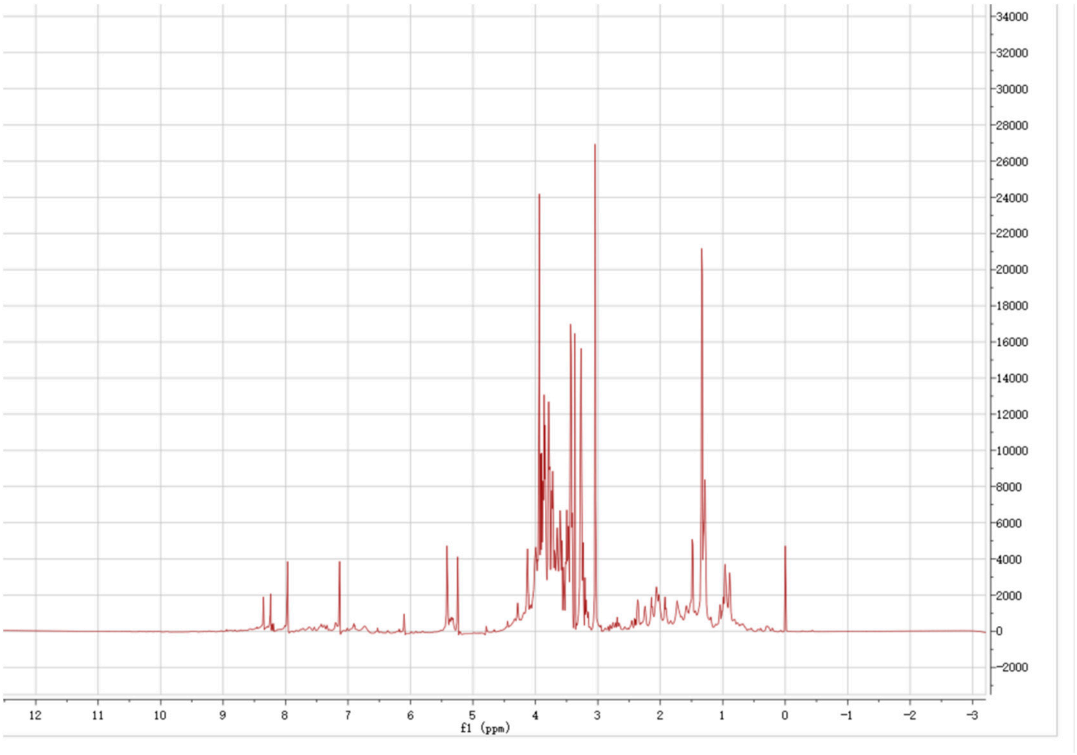

Figure S3. Representative 600MHz ^{1}H NMR spectra of zebrafish liver samples. The y-axis represents signal intensity in arbitrary units.
